# Scale-dependency of the global mean surface temperature trend and its implication for the recent hiatus of global warming

**DOI:** 10.1038/srep12971

**Published:** 2015-08-11

**Authors:** Yong Lin, Christian L. E. Franzke

**Affiliations:** 1 National Marine Environmental Monitoring Center, State Oceanic Administration, Dalian, 116023, China; 2 Meteorological Institute and Center for Earth System Research and Sustainability, University of Hamburg, Hamburg, Germany

## Abstract

Studies of the global mean surface temperature trend are typically conducted at a single (usually annual or decadal) time scale. The used scale does not necessarily correspond to the intrinsic scales of the natural temperature variability. This scale mismatch complicates the separation of externally forced temperature trends from natural temperature fluctuations. The hiatus of global warming since 1999 has been claimed to show that human activities play only a minor role in global warming. Most likely this claim is wrong due to the inadequate consideration of the scale-dependency in the global surface temperature (GST) evolution. Here we show that the variability and trend of the global mean surface temperature anomalies (GSTA) from January 1850 to December 2013, which incorporate both land and sea surface data, is scale-dependent and that the recent hiatus of global warming is mainly related to natural long-term oscillations. These results provide a possible explanation of the recent hiatus of global warming and suggest that the hiatus is only temporary.

Global warming has been widely studied over the past few decades^1^,^2^. While it has unequivocally been shown that global warming is human induced, natural climate variability still plays a significant role and likely can still overshadow the global warming trend. For instance, the hiatus of global warming (the leveling off of the global mean temperature increase) over the past 15 years is likely to be due to natural climate variability counteracting the anthropogenic induced global warming trend^1^. Recent studies provide evidence that global warming trends are nonlinear^3^. These decadal scale variations have been attributed to an increased heat transfer to the deep sea^4^ counteracting the anthropogenic induced surface warming. Furthermore, the time series analysis of extreme maximum temperatures shows that global warming continued over the recent decades^5^. This provides further evidence for the continuing impact of human induced global warming on local and regional scales if not globally.

The Selection of temporal scales is essential for climate change studies, because different scales used for the same system can produce very different results. For instance, in the study of large-scale forest harvesting on the hydrology in the willow watershed of central British Columbia, Lin and Wei reported that forest harvesting significantly increased the mean and peak flows at the annual scale, whereas the mean and peak flows in the summer and winter periods (corresponding to the seasonal scale) were not significantly affected^6^. The recent hiatus of global warming is perhaps related to the problem with the selection of time scales in addition to the parameters used, as shown by Seneviratne *et al.* who found that the global warming trend continued over the recent decades when maximum extreme temperatures rather than average temperatures were used^5^.

In most previous studies of global warming, the used temporal scales (usually a single scale) are arbitrarily selected and typically do not correspond to the intrinsic scale of the studied geophysical phenomenon or process in itself. Despite the evidence for a continuum of time scales^7^,^8^, there are intrinsic scales for geophysical and ecological processes (or patterns). According to Levin, there is no single natural scale at which ecological phenomena should be studied and systems generally show characteristic variability over any range of spatial, temporal and organization (e.g. individual, population, ecosystem/community, landscape, region and globe) scales^9^. Given that patterns that are unique to a range of scales (intrinsic scales in our opinion) have unique causes and biological consequences^9^, we should address the global surface temperature’s evolution and the hiatus over the recent two decades at the appropriate intrinsic scales.

Some natural cycles, like the daily and seasonal cycles, are well known to scientists. However, some cycles or quasi-cycles, especially on lower frequencies, are either unknown to us or even ignored in data analysis. In this case, we are very likely to mistake the downward part of a cycle as an externally forced trend. To complicate matters further, there is also a continuous background spectrum on all time scales^7^,^8^. However, because natural quasi-oscillations like the Atlantic Multi-decadal Oscillation (AMO) and El Nino (ENSO) play important roles in the temperature evolution, it is important to disentangle their contribution from the anthropogenic forced warming trend. When we discuss the hiatus of global warming in the recent decades, we should address the issues of both long term and short term evolution patterns (oscillations) driven by natural forces.

The mismatch between the intrinsic natural scales and data analysis scales might present us with misleading or spurious trends. The downward part of a long-term natural oscillation can give us a picture of the global warming pause. Is this the cause of the recent hiatus? In addition, temperature change rates, commonly used in global warming studies, are sensitive to outliers in temperature time series resulting from small-scale variability or events. Is this small-scale variability one of the reasons for the hiatus of global warming? Here we attempt to answer these questions with the Multi-Resolution Analysis (MRA) and the wavelet power spectrum analysis of global surface temperature anomaly (GSTA) time series.

## Data and Methods

Here we use the monthly mean GSTA (relative to the 1961–1990 mean) time series, which incorporates both land surface air temperature and sea surface temperature data and spans the time period from January 1850 to December 2013, to study the global mean surface temperature trend. In spite of the low quality of the pre-1880 data, the inclusion of these data is very useful for understanding the GSTA long-term evolution. For more details about the GSTA data please refer to http://cdiac.ornl.gov/trends/temp/jonescru/jones.html.

Wavelet analysis provides a systematic way to obtain information that is not readily available in the raw data. Wavelet analysis has two main applications, namely, cleaning (noise and blur reduction) and signal analysis (to determine how the frequency content of a signal evolves over time), which are based on discrete wavelet transforms (CWT) and continuous wavelet transforms (DWT), respectively. The DWT based MRA decomposes the original (temporal or spatial) series into a low frequency component (or approximation part, usually regarded as the trend) and a high frequency component (or detail part, usually regarded as noise or fluctuation) and this process can be repeated for the approximation part at various levels, producing a series of overall pictures of the geophysical time series at various scales and detail parts corresponding to various decomposing levels or scales as well. MRA undoubtedly increases the chance of scale match between intrinsic scales of a natural process or pattern and the data analysis scale in that many scales rather than a single scale are used in multi-resolution analysis. The continuous Wavelet transform, which decomposes a time series into time-frequency space, can be used to determine both the dominant modes of variability and how those modes vary in time by means of Wavelet power spectrums^11^. These two functions of wavelet analysis are useful in this study in that they can help us gauge if the recent hiatus affects the overall global warming trend when observed at bigger temporal scales and if the hiatus is related to the existence of some small-scale (high frequency) natural oscillations (e.g. ENSO with a return period of 2–7 years) in the last two decades.

Wavelet transformation coefficients or wavelet powers (defined as the square of wavelet transform coefficients) yield information as to the correlation between the wavelet (at a certain scale) and the data series or array (at a particular location). The large wavelet power value at a given scale (s) and at a particular time location (t) means that the oscillation at the frequency related to that scale (s) exists at the time period centered on the time location (t). As we are interested in the questions whether short-term natural variability causes the hiatus and whether the hiatus affects the long term warming trend, cleaning and the signal analysis, the two main functions of wavelet analysis are both used in this paper.

In the process of wavelet transforms, whether discrete or continuous, the distortion problem with the wavelet transform coefficients at the beginning and end of the data series arises due to the finite time series length^11^,^12^. Given that the recent hiatus occurs just at the end part of the GSTA time series and its duration is at least about 15–20 years, the influence of border distortions on the analysis result needs to be carefully considered. To deal with border distortions for MRA, the signal extension is made on the left side with the half-point symmetry method, producing an extended time series of 2048 months. The resultant GSTA time series is first reversed in order and the reversed time series data are used for MRA using the Daubechies 5 (db5) wavelet, these approximations at various decomposition levels are reversed again for the analysis of the GSTA evolution dynamics. The Approximations of the GSTA time series at the levels of 8, 7, 6 and 5, corresponding to the scales of 256 months, 128 months, 64 months and 32 months, respectively, are plotted for our GSTA trend study. The main reason for both the back-and-forth reversing of time series and left side extension is that we want to avoid the spurious or unreliable trend for the hiatus period. As the hiatus period is just on the right side of the time series and of most interest to us, the convolution with extended data on the right side for MRA should undoubtedly be avoided. As far as the continuous wavelet transform is concerned, wavelet transform coefficients used to calculate wavelet powers at the end points are usually distorted due to convolution with no further data points. To reduce this problem, the whole-point symmetric extension mode at both sides is used to produce an extended time series which have 4096 rather than 2048 data points in this study. The resultant extended time series is then used to calculate wavelet transform coefficients and (rectified) wavelet powers using the Mexican hat wavelet. The availability of 1064 extended data points for both sides is expected to reduce the boundary distortion resulting from limited time series (1968 data points here) since even the cone of influence (COI) of the extended time series for the maximum scale of 240 months in this study does not cover the original data points according to the formula for the size of COI provided by Torrence and Compo (1998)^11^.

According to Liu *et al.*^10^, the traditional wavelet power spectrum should be rectified by a factor related to the scale and the orthonormal wavelet function is preferred to avoid bias and to be physically meaningful. However, the Mexican hat wavelet, a non-orthonormal type of wavelet function is used in this paper to calculate rectified wavelet powers with reference to Liu *et al.*^10^. The main reason for the usage of the Mexican hat wavelet is that this wavelet function is less affected by boundary distortion and more appropriate for the study of quasi-periodic features^11^,^12^. The significance test for the wavelet analysis is very important due to the simple fact that some apparent patterns (e.g. Peaks) show up even if the analyzed signal is pure noise^13^. To deal with this issue, a significance test for wavelet power spectrums is conducted with red noise as background spectrums according to Torrence and Compo^11^. As the used wavelet function (Mexican hat) is not complex, the formula Eq. (18) in Torrence and Compo (1998)^11^ is changed accordingly, that is, the 1/2 is removed and the degree of freedom for chi-square distribution is set to 1 rather than 2. It is found that the monthly GSTA time series has serial correlations of 0.909 (α_1_) and 0.882 (α_2_) at the lags of 1 and 2, respectively. These value are then used to calculate the value for α in Eq. (16) in Torrence and Compo (1998)^11^.

The relationship between the Wavelet scale (s) and the equivalent Fourier period (λ) is very useful to get the information on the duration of the oscillations determined by wavelet powers. According to the formula in table 1 of Torrence and Compo (1998)^11^ for wavelet DOG (derivative of a Gaussian), the conversion coefficient from wavelet scale (s) to equivalent Fourier period (λ) for the Mexican hat wavelet (m = 2) is 3.97. That is, the period of the oscillation determined by the Mexican hat wavelet at the scale s is about 4 s, which is used to estimate the duration of oscillation determined by the wavelet power spectrum using the Mexican hat wavelet in our study.

## Results

**Figure 1:**
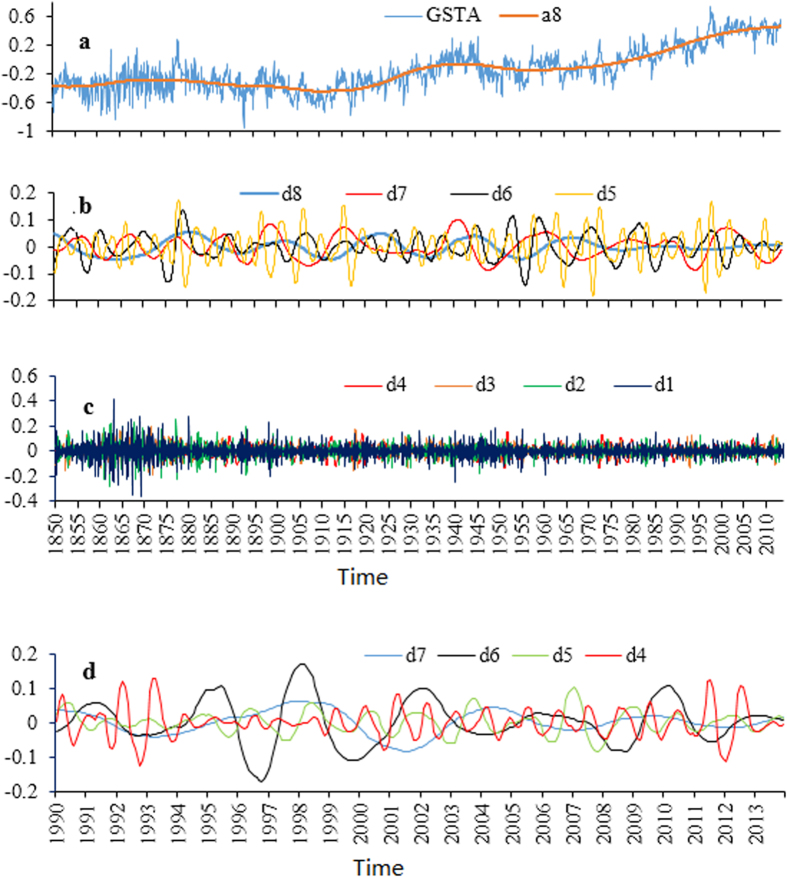
Monthly global surface temperature anomaly (GSTA) time series from January 1850 to December 2013 and its approximation (a8) at the level of 8 (**a**) and detail parts of different levels (d8, d7, d6 and d5 in **b**; d4, d3, d2 and d1 in **c**), namely, fluctuations of different frequencies, based on multi-resolution analysis using wavelet function Daubechies 5. Detail parts at the levels of 7, 6, 5 and 4 for the period of 1990–2013 are shown in **d**.

The global temperature evolution is composed of many oscillations of various frequencies which are the result of the nonlinearity of the climate system and the many periodic and aperiodic natural forces. The existence of such oscillatory components of high frequency (usually related to noise, Fig. 1b–d) often overshadows the lower frequency modes (trends or patterns) which are of interest to us (Fig. 1a). This highlights the importance of noise and blurs cleaning by MRA. Seen from Fig. 1b, the recent hiatus which started in around 1999 is mainly associated with the crests and troughs in the components of d7, d6 and d5 related to time scales of 128 months, 64 months and 32 months, respectively. The low fluctuation of component d8 (Fig. 1b) and the obvious downward change from 2002 to 2011 in component d7 (Fig. 1b,d) are the main causes of the recent hiatus of global warming. In addition, the coexistence of d7 crest and d6 trough in around 2004 and that of d7 trough and d5 crest in around 2012 contribute to the leveling off of the GST increase in the recent two decades (Fig. 1b,d). The much smaller peak (0.020 °C) in around 2013 when compared with its precedent peak (0.107 °C) in around 2010 for d6 suggests that the small-term variability is likely responsible for the recent global warming hiatus (Fig. 1b,d). Short-term oscillations with intervals below 12 months shown in Fig. 1c (d3, d2 and d1) are not supposed to affect previous hiatus studies because the scales used there are usually at annual or longer time scales.

**Figure 2:**
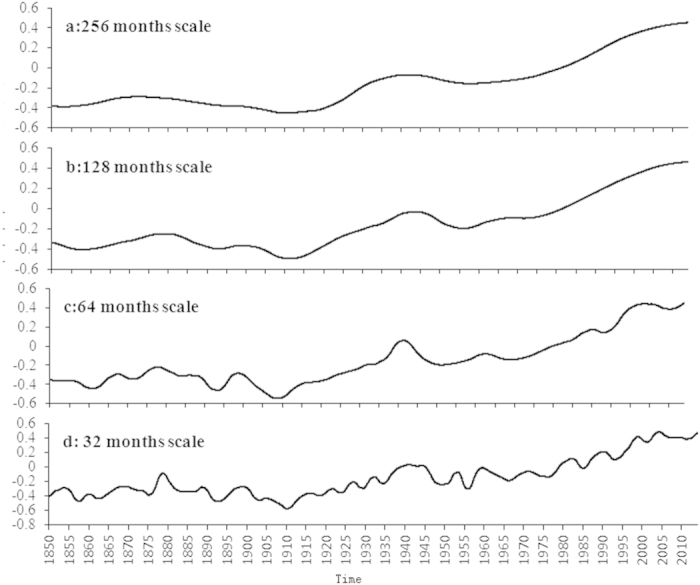
Trends of global surface temperature evolution observed at the scales of 256 (**a**), 128 (**b**), 64 (**c**) and 32 (**d**) months according to the multi-resolution analysis of monthly GSTA time series from January 1850 to December 2013.

**Figure 3:**
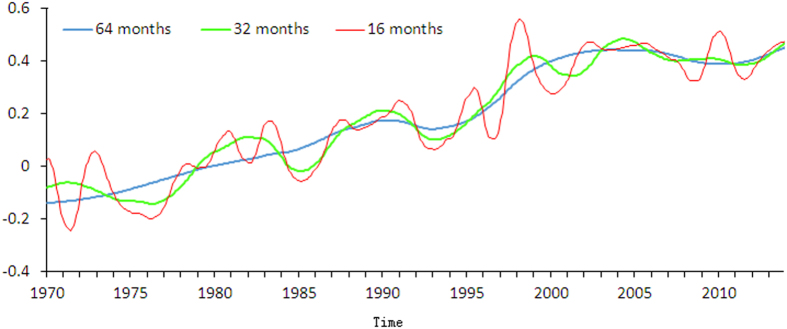
Trends of global surface temperature evolution observed at the scales of 64, 32 and16 months according to the multi-resolution analysis of monthly GSTA time series from January 1970 to December 2013.

MRA is a useful tool to systematically extract the structure of the GST evolution pattern at different scales (Fig. 2). We find evidence for 3 long term oscillations with durations ranging from 45 to 80 years or longer. Our results suggest that the GSTA can be expected to peak in the near future when we extrapolate the observed time series at the scales of both 256 months and 128 months (Fig. 2a,b). The expectation of the GST change rate slowing down before and after this peak suggests that the recent hiatus is related to a long term oscillation. The Atlantic Multi-decadal Oscillation (AMO)^14^ and ocean heat uptake^15^ which have a similar frequency might play a role on such time scales. In addition, the fact that GSTA values in the years of 2000, 2008 and 2011 are much lower than their surrounding values at the time scale of 16 months suggests that the recent hiatus is related to short-term events (Fig. 3).

Although the GSTA evolution patterns at the scales of 256 months and 128 months (Fig. 2a,b) are helpful to explain the recent hiatus, the global warming hiatus pattern is not visible at these two scales. In contrast, the hiatus is very obvious when observed at the scales of 64 months and 32 months (Fig. 2c,d), suggesting that the consideration of the scale-pattern issue is essential for global warming studies. In addition, it is shown that there were several hiatuses of warming or cooling periods in the GSTA evolution pattern, for instance, for the period of 1870–1914 (Fig. 2a) and the period of 1944–1957 (Fig. 2b). The hiatus period (for instance, 1896–1914 in Fig. 2a) at a given scale (256 months) shows obvious oscillation pattern, which is composed of a downward part (1896–1910) and an upward part (1910–1914), when observed at a finer temporal scale (here at 128 months scale, Fig. 2b), highlighting the importance of scale selection for the study of global warming. The comparison of the GSTA evolution patterns at finer scales (Fig. 3) for the period of 1970–2013 also shows similar results.

The recent low-frequency oscillation with an upward part lasting more than 30 years and likely peaking in 2013 (Fig. 2a,b) suggests that a decreasing trend over the next 30 years is possible. However, in the case that the recent oscillation is similar to that from 1912 to 1956 (Fig. 2b), a new global warming trend will occur much earlier, thus ending the current hiatus. In fact, it is clear that the downward periods of oscillations for global surface temperature evolution are much shorter than the upward periods as shown in Fig. 2b–d. However, this can only hold if anthropogenic global warming does not and will not affect these natural climate system oscillations in the future.

The hiatus periods of 1990–1994 and 2004–2012 at the scale of 64 months show obvious oscillation patterns when observed at the scale of 32 months (Figs 2 and 3). This suggests that the occurrence of the global warming slowdown from 1999 onwards does provide evidence that natural fluctuations can still impact global mean temperature and temporarily counteract anthropogenic global warming. The evolution patterns of GSTA at various scales clearly show that the GSTA values in the recent hiatus period are still much higher than those before 1900 when human influences on the global scale were much smaller in spite of a slow-down of the GSTA increase rate. The increase of GSTA from −0.495 °C in 1912 to 0.462 °C in 2013 observed at the scale of 128 months is not as small as it appears because this long-term (about 10 years, Fig. 2b) averaged GSTA value is also globally averaged. Even the temperature in the recent decade is 5.0 °C higher than that in a given 10-year period before the industrial revolution, the temperature difference between pre-industrial and post-industrial revolutions at the century scale may be less than 1.0 °C as a result of long-term averaging. The warming (or temperature change) amplitude still gives strong support for anthropogenic global warming.

**Figure 4:**
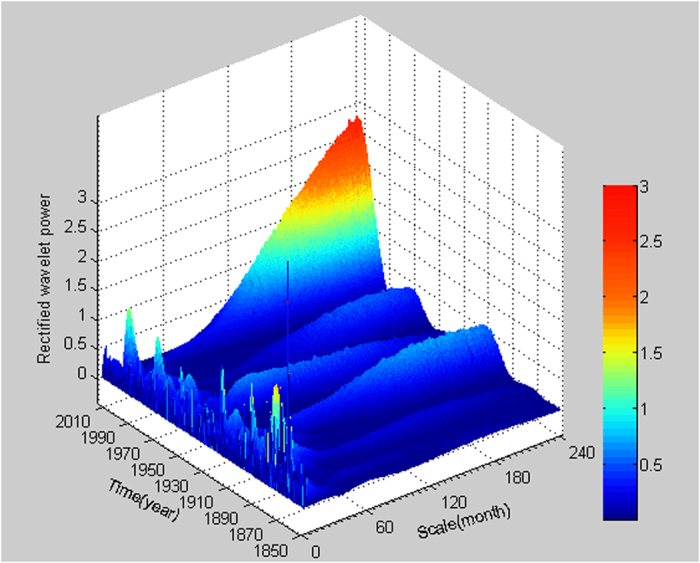
Rectified wavelet power spectrums of normalized GSTA time series spanning from January 1850 to December 2013 using wavelet Mexican hat.

**Figure 5:**
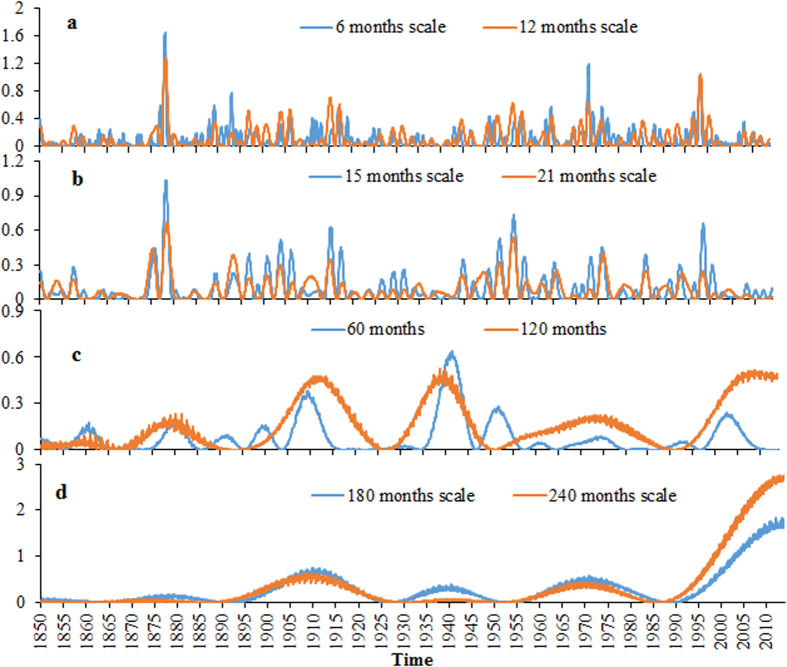
Rectified wavelet spectrums of normalized GSTA time series at the scales of 6, 12 (**a**), 15, 21 (**b**), 60, 120 (**c**), 180 and 240 months (**d**).

The wavelet power spectrum map for a time series can be used to determine the locations where the periodic components of various frequencies occur. The high wavelet power at a given scale and a given time location means that the oscillation of the frequency related to this scale exists at this time location. Traditional wavelet powers have the problem of underestimating the wavelet power values at small scales and might not be physically meaningful, affecting the determination of dominant oscillation modes^10^. Therefore, the usage of the rectified wavelet power spectrum which corrects this problem is essential. The rectified wavelet power spectrum for the GSTA time series is shown in Fig. 4. It is found that the hiatus of the global surface temperature increase in recent decades is mainly related to long-term oscillations as the rectified wavelet power values in the hiatus period at large time scales are much bigger than those at small time scales. The rectified wavelet powers at some small scales and large scales are shown in Fig. 5 for a better comparison. It is clear that the wavelet powers at the scales of 180 and 240 months are much bigger than those at other scales in the hiatus period (Fig. 5a–d). This suggests that an oscillation of about 80 years might have caused the recent hiatus because the conversion coefficient from scale to Fourier period for wavelet powers based on the Mexican hat wavelet is 3.97. This gives us some information on the length of the hiatus period. The fact that the wavelet powers at the time scales of 180 and 240 months in the period of 1999–2013 are much bigger than those at the same scales in all other periods, suggests that the recent hiatus is special to some extent and that the hiatus is only temporary (Fig. 5d).

**Figure 6:**
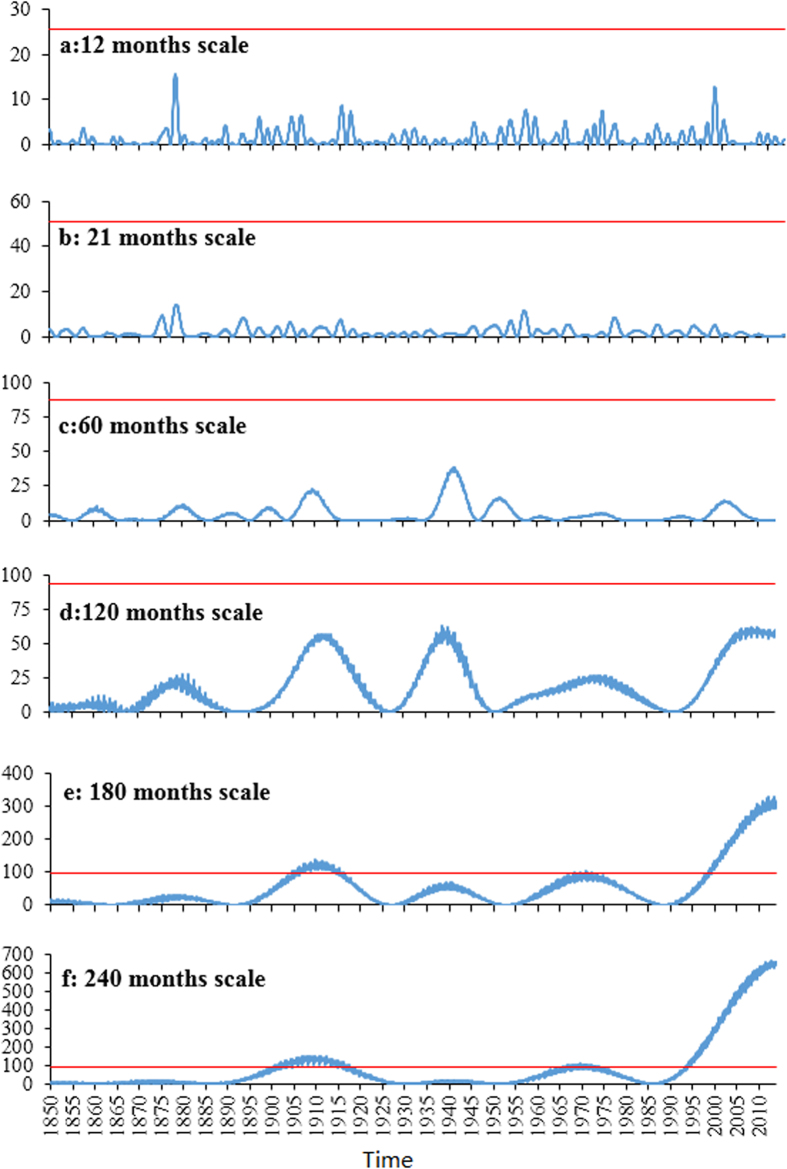
Normalized wavelet power spectrums of GSTA time series at the scales of 12 (**a**), 21 (**b**), 60 (**c**), 120 (**d**), 180 (**e**) and 240 (**f**) months, respectively. The red lines are the 90% confidence levels for the corresponding red noise spectrum.

Statistical tests for the peaks of wavelet power of GSTA against a red noise spectrum are also conducted. Such tests are very important because some apparent patterns (e.g. peaks) can arise even if the analyzed signal is pure noise^13^. It is found that all the oscillations of the periods of about 2–7 years (corresponding to time scales of 6, 9, 12, 15, 18 and 21 months) occurring in the recent hiatus period fail to pass red noise based statistical test, suggesting that short-term variability is not the cause of the recent hiatus (only 2 and 7 years shown here in Fig. 6a,b). In contrast, the peaks for longer term oscillations of the duration longer than 60 years in the recent hiatus (Fig. 6e,f) are statistically different from red noise, suggesting that long-term oscillations are responsible for the recent hiatus. According to Ge^13^, theoretically meaningful 5% probability is too stringent since the signal-to-noise ratio in observed data is often low. We use 90% rather than 95% as confidence level for statistical tests in this study. Despite that the curves in Fig. 6 show similar information as those in Fig. 5, wavelet power spectrums shown in Fig. 6 are necessary because statistical tests against red noise are based on (traditional) wavelet powers rather than rectified wavelet powers. To our knowledge, the rectified wavelet powers based statistical test method against red or white noise is not available in scientific documents.

According to NASA, the EI Nino pattern of climate variability that has historically coincided with a slowing in global warming is perhaps a major cause for the recent hiatus (http://washington.cbslocal.com/2014/08/06/nasa-climate-scientist-explains-15-year-global-warming-hiatus/). However, our statistical results at the scales of 6, 9, 12, 15, 18 and 21 months (corresponding to the periods of 2, 3, 4, 5, 6 and 7 years) do not support this claim. In contrast, the role of long-term oscillations of long duration in the recent hiatus of global warming is statistically supported. The increased heat transfer to the deep sea with a duration of about 30 years^4^ (just half of an oscillation of 60 years) may be the cause of the upward trend in Fig. 2b and the peak for the rectified wavelet power at the scale of 180 and 240 months (corresponding to the oscillations of 60 and 80 years in duration) in Fig. 6e,f.

## Conclusions

The hiatus of global warming over the recent two decades has been widely discussed. Numerous explanations have been given for the hiatus, but none of them addressed this issue from the point of view of the relationship between pattern and time scale. Traditional climate change studies are usually conducted at an arbitrary selected scale rather than the intrinsic ones unique to the processes of interest (global temperature evolution here). The pattern of the GST evolution is scale dependent and the same GST evolution process when observed at different scales will produce different results^16^. Our results suggest that the recent hiatus is due to natural fluctuations imposing a decreasing temperature trend and, thus, temporally overshadowing the global warming trend. This means that the global warming hiatus is temporary. Our multi-scale analysis of GST time series spanning from January 1850 to December 2013 shows that the hiatus is associated with long term oscillations whereas the role of small-scale variability in the recent hiatus is not statistically significant. The fact that the hiatus period is scale dependent highlights the importance of scale selection for climate change studies. In spite of the low warming rate over the recent two decades, the temperatures in the hiatus period are still much higher than those in the pre-hiatus period, suggesting that anthropogenic global warming still exerts a strong signal and is worth world-wide concern.

## Additional Information

**How to cite this article**: Lin, Y. and Franzke, C. L. E. Scale-dependency of the global mean surface temperature trend and its implication for the recent hiatus of global warming. *Sci. Rep.* **5**, 12971; doi: 10.1038/srep12971 (2015).
